# Protein Conformational Changes in Breast Cancer Sera Using Infrared Spectroscopic Analysis

**DOI:** 10.3390/cancers12071708

**Published:** 2020-06-27

**Authors:** Hemendra Ghimire, Chakravarthy Garlapati, Emiel A. M. Janssen, Uma Krishnamurti, Gengsheng Qin, Ritu Aneja, A. G. Unil Perera

**Affiliations:** 1 Department of Physics and Astronomy, Georgia State University, Atlanta, GA 30303, USA; hghimire1@student.gsu.edu; 2 Department of Biology, Georgia State University, Atlanta, GA 30303, USA; cgarlapati1@student.gsu.edu (C.G.); raneja@gsu.edu (R.A.); 3 Department of Pathology, Stavanger University Hospital, Stavanger NO-4068, Norway; emilius.adrianus.maria.janssen@sus.no; 4 Department of Pathology, Emory University School of Medicine, Atlanta, GA 30322, USA; umakrishna@emory.edu; 5 Department of Mathematics and Statistics, Georgia State University, Atlanta, GA 30303, USA; gqin@gsu.edu; 6 Center for Diagnostics and Therapeutics, Georgia State University, Atlanta, GA 30303, USA

**Keywords:** ATR-FTIR, infrared spectroscopy, spectral deconvolution, serum, protein secondary structure, breast cancer biomarkers

## Abstract

Protein structural alterations, including misfolding and aggregation, are a hallmark of several diseases, including cancer. However, the possible clinical application of protein conformational analysis using infrared spectroscopy to detect cancer-associated structural changes in proteins has not been established yet. The present study investigates the applicability of Fourier transform infrared spectroscopy in distinguishing the sera of healthy individuals and breast cancer patients. The cancer-associated alterations in the protein structure were analyzed by fitting the amide I (1600–1700 cm^−1^) band of experimental curves, as well as by comparing the ratio of the absorbance values at the amide II and amide III bands, assigning those as the infrared spectral signatures. The snapshot of the breast cancer-associated alteration in circulating DNA and RNA was also evaluated by extending the spectral fitting protocol to the complex region of carbohydrates and nucleic acids, 1140–1000 cm^−1^. The sensitivity and specificity of these signatures, representing the ratio of the α-helix and β-pleated sheet in proteins, were both 90%. Likewise, the ratio of amides II and amide III (I_1556_/I_1295_) had a sensitivity and specificity of 100% and 80%, respectively. Thus, infrared spectroscopy can serve as a powerful tool to understand the protein structural alterations besides distinguishing breast cancer and healthy serum samples.

## 1. Introduction

Breast cancer (BC) is the most common invasive cancer among women worldwide [1]. The international agency for research on cancer (IARC) reports that BC comprises 22.9% of invasive cancers in women [1,2]. At present, personal inspection and imaging remain the preferred methods for screening asymptomatic women for BC. Nonetheless, the gold standard mammography entails high costs, is not available in all medical centers, and has a low sensitivity in young women and in the dense breast. Furthermore, BC typically produces less to no symptoms when the tumor is small and easily treatable [3]. The established mammography screening may miss up to 20% of underlying breast cancers [4]. It may also lead to a 30% rate of overdiagnosis and may increase unnecessary surgical procedures and patient anxiety [5]. These limitations have led to the investigation of blood-associated protein markers [6] that can be used for BC screening before mammography. The feasibility of markers such as CA15-3, HSP90A, and PAI-1 for early prognosis [7] is still unclear. It is thus critical to explore potential new markers that can help with the early detection of BC. Our study focuses on evaluating the feasibility of Fourier transform infrared (FTIR) spectral discrimination on serum from healthy controls and BC patients using spectral deconvolution.

### 1.1. Biomedical Application of FTIR Spectroscopy

FTIR spectroscopy is a powerful analytical tool that can be used to provide insight into the composition, structure, and interaction of constituent molecules within biological solutions [8,9]. Changes in the characteristics of biological fluids which often occur in disease can be detected in spectral data and have emerged as a robust tool in clinical studies over the past few years [10]. An increasing number of studies have demonstrated the effectiveness and promising application of this technique in several biological sciences [11]. The most widely used peak frequencies and their assignments during biochemical studies are also summarized [12]. Standard protocols for the measurement of diagnostic mediums using FTIR spectroscopy [13] and spectral analysis techniques are well established [14]. However, examining the differences between the FTIR spectra has proven to be challenging due to the complexity of the biological constituents, which have different vibrational modes [15,16]. Therefore, in FTIR studies sophisticated spectral analysis techniques are employed to overcome these inherent spectral interpretation challenges caused by highly overlapping absorbance peaks [15].

Curve fitting (spectral deconvolution) is one of the analytical methods that can be used to improve the resolution of complex spectra and has successfully been used to determine proteins’ secondary structure by infrared spectrometry [14]. Importantly, resolution improvement allows for a more precise analysis by increasing the confidence of results when FTIR spectrometry is used in clinical applications. However, the possible clinical application of spectral fittings in the comparison of serum samples from healthy individuals and BC patients has not [17] been extensively explored.

Moreover, the protein regulation [18], expression [19], and profiling [20] of tissues are commonly used as indicators for the diagnosis, treatment, and prognosis of various stages of BC [21]. FTIR spectrometry has also been successfully applied to commonly used diagnostic material, such as blood components [21,22,23,24], breast tissue [17,25], hair [26,27], and other biological samples [28], to discriminate breast cancer samples [17,22,23,24,25,26,27]. Contrastingly, the applicability of the curve fitting technique while discriminating the infrared spectra of control and BC sera samples has not been well understood [29]. Spectral fitting using characteristic Gaussian Function Energy Bands (GFEB) improves the resolution and eases the inherent infrared spectral analysis difficulties involving highly overlapping absorbance peaks [14]. In fact, a key element appears to be missing—the details of GFEB that have been attributed to the specific functional groups present in sera and their BC-induced changes.

Additionally, body fluids, including blood-components, are considered as precious and ideal diagnostic mediums of clinical biomarkers [30] owing to the advantages of minimal invasiveness, low cost, and rapidity of sample collection and processing. The assessment of BC-associated changes in the protein secondary structures of body fluids will thus be an emerging interest over the existing histopathological examination of the breast biopsy materials. Alterations in the biochemical composition of the serum could reflect changes in physiological states due to BC, enabling early disease diagnosis and treatment [31].

### 1.2. Protein Conformational Studies Using FTIR Spectroscopy

X-ray crystallography and nuclear magnetic resonance (NMR) have been widely used to examine the structure of proteins and other biological macromolecules [32]. Despite the fact that they have been successfully used in biochemical studies over the years, when it comes to the high-resolution analysis of protein structure and function, the use of these complementary spectroscopic methods is hindered by the need for sampling protocols [33] and sophisticated data analysis tools. The X-ray diffraction technique requires a well-ordered crystal [33], while the use of NMR spectroscopy is limited to small proteins [34]. Data analysis protocols for these techniques are also complex, complicating the interpretation of the results. These limitations have led to the development of alternative methods for determining protein structures.

FTIR spectroscopy is one alternative method that can be used for protein secondary structure analysis [35,36,37]. In previous reports, the FTIR spectroscopic investigation of protein secondary structures [38] in BC patient serum samples was validated by several other analytical techniques [39], such as X-ray, NMR [40], and Circular Dichroism spectra (CD) [41]. The FTIR technique has also been tested with various sample types and conditions, including living cells [42], aqueous media [43], hydrogen deuteration [44] in serum [45], dehydration [46], and the heat-induced [47,48] denaturation of serum. Additionally, spectral deconvolution [41] has been employed to diagnose or monitor various ailments, including prostate cancer [49], lymphoma [50], melanoma [50], Alzheimer’s disease [51], Parkinson’s disease [52], colitis [37], and scrapie [53]. Moreover, this method has been successfully used to study protein–protein interactions [54]; the structure of calcium-binding proteins [55]; and the understanding of the uses and misuses of techniques [56], their optimizations [57], and instrumental improvisations [58]. The protein structure as well as protein conformational changes [59], structural dynamics, and stability have also been successfully determined using second derivative curves [60]. All in all, FTIR spectroscopy has emerged as a powerful tool to study protein secondary structures and can be clinically useful in the early diagnosis of diseases.

In the present proof-of-concept pilot study, we have used FTIR spectral discrimination using curve fitting to obtain the best fit that reflects protein conformational changes in the serum samples of BC patients. The curve fitting technique is also elaborated on in the complex spectral region of carbohydrates and nucleic acids, 1000–1140 cm^−1^ [12,61,62]. By deconvoluting these regions of experimental spectra with the corresponding GFEB of various biological components, the differentiating signatures of controls and cancerous spectra were determined. Other infrared spectral markers, such as the peak positions of the absorbance curves and spectral signatures, such as the ratio of absorbance values in amide II to amide III bands, are also considered for discrimination. Statistical analysis is further performed in these identifying spectral signatures to understand the discriminating potential. Herein, the accepted scientific premise is that the BC-associated genetic alteration in serum is reflected in the complex region of nucleic acids, including deoxyribonucleic acids (DNA) and ribonucleic acids (RNA) [61,62]. Therefore, our discussion also includes the possible application of genetic and proteomic molecular mapping in serum samples via FTIR spectroscopy for the earlier detection of BC. We have incorporated statistical measures, holistically evaluated the biochemical mapping of proteins structures and circulating nucleic acids components by using infrared spectral deconvolution. A unified fitting protocol for all the samples and a potential prototype applicable in the clinical domain is also presented. These findings go beyond the earlier study [29], providing spectral signatures with higher sensitivities and specificities. Similarly, the implementation of optimized experimental and data analysis protocols and quantification of the spectral signatures by scrutinizing molecular entities rather than relying entirely on wider spectral ranges are improvements over the earlier study [22].

## 2. Results

Using the absorbance spectral data of serum samples (using n = 10 for each BC and control), we investigated the applicability of FTIR spectroscopy to discriminate between the control and cancer sera. The attenuated total reflectance (ATR) sample mode of FTIR spectroscopy was used, and the discrimination between the control and test groups was conducted using various data analysis techniques. The investigation involves multivariate analysis, *p*-value calculation, and quantification by spectral deconvolution and is followed by a statistical analysis.

### 2.1. Principal Component Analysis (PCA)

**Figure 1:**
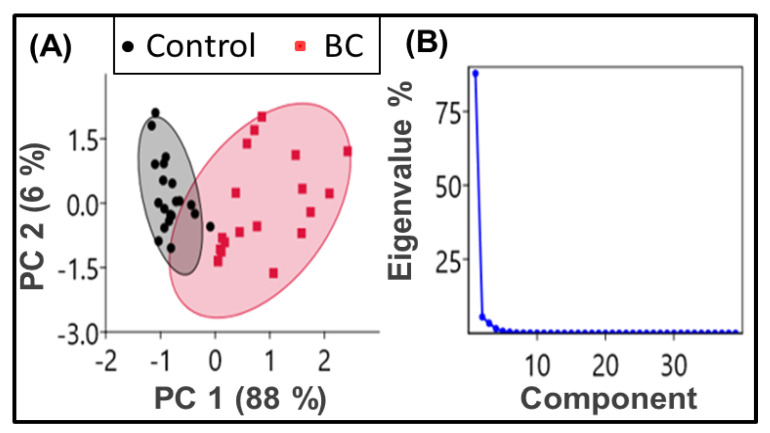
Principal Component Analysis (PCA) of the second derivatives curves of the FTIR absorbance spectra. (**A**) PCA scores plots (PC1 × PC2) with a 95% confidence ellipse. The data related to the control groups (black dots enclosed by a black shaded ellipse) and breast cancer (BC) (red dots surrounded by a red-shaded ellipse) are clustered together with different magnitudes and directions. (**B**) Scree plot of eigenvalues showing the percentage variance of components one and two is significant compared to the others.

PCA, a useful statistical analysis [63], is first performed to explain the holistic evaluation of protein structural content variations reflected in amides (amide I and II region, 1480–1600 cm^−1^). Herein, each of the 10 samples is measured twice (measurement replicates) to obtain 20 spectral data of BC and 20 of the control. Using the “PAST (PAleontological STatistics) 4 - the Past of the Future” software and the vector normalized second derivative curve of the absorbance spectra within 1480–1600 cm^−1^ as input data variables, we analyzed the variance-covariance matrix with the pairwise exclusion of missing values to get the component plots. The output of the component plot with 95% ellipses shows (Figure 1A) a clear separation between each studied group. The scatter plot of PC1 (variability 88%) and PC2 (variability 6%) shows that the data related to the control and BC groups are clustered together with different magnitudes and directions. Figure 1B is the scree plot, showing that the total variance presented by PC1 and PC2 are significant. These findings from the PCA analysis of the amide bands have led us to investigate the spectral signatures useful in the clinical domain.

### 2.2. Discrimination of Average Absorbance

**Figure 2:**
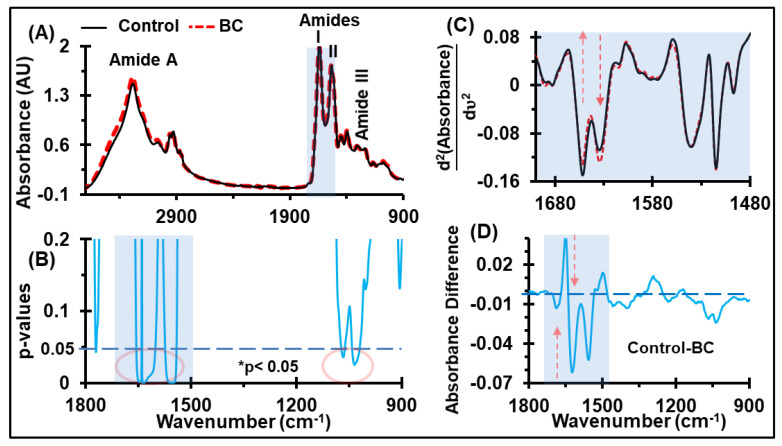
Identification of discriminatory bands. (**A**) Ensemble averages of normalized serum spectra derived from control, n = 10, and BC, n = 10. This wider range of spectra is presented to show the quality of spectra, which overcomes the noise and atmospheric contamination, while measuring them at a resolution of 4 cm^−1^. (**B**) Corresponding Student t-test p-values for the control and BC. (**C**) The second derivative absorbance spectra is confined to the amide-I region, covering 1600–1700 cm^−1^. (**D**) Difference between the absorbance spectra of the control and BC (shown in Figure 1A), indicating the up- and down-regulation of proteins, carbohydrates, and nucleic acids in the serum of breast cancer patients.

The average of the normalized absorbance spectra for both the control and BC sera that includes the fingerprint region of the biological functional groups (lipids, proteins, nucleic acids, and carbohydrates) is shown in Figure 2A. Solely by looking at the FTIR spectra, it is difficult to discriminate between the absorbance of the functional components of the two groups. However, the comparison of the absorbance spectra between the two groups using a Student’s *t*-test (with two-tailed unequal variance) revealed the discriminating potential at the amide regions (1541–1656 cm^−1^) and mixed regions of carbohydrates and nucleic acids (1018–1076 cm^−1^), as highlighted by the red ellipses in Figure 2B (*p* < 0.05). The prominent discriminatory regions include C=O/C-N stretching, N-H bends in amides, RNA/DNA nucleotides, and C-O vibrations of carbohydrates [64], as reported in previous studies [22]. Previous studies using a principal component analysis-linear discriminant analysis (PCA-LDA) of the FTIR spectra have shown that healthy and cancerous serum samples have different characteristics [22].

The molecular assignments of major spectral bands showing discrimination between the control and BC with higher significance (i.e., *p*-values < 0.05), are also presented in Table 1. These are the bands originating from the amides of protein, carbohydrates, and nucleic acids. The amide vibrations are mainly arising from the C=O stretching vibration, with minor contributions from out-of-phase C-H stretching vibrations, C-C-N deformation, and N-H in-plane bending [14]. Similarly, the mixed regions of carbohydrates and nucleic acids result from the C-O/C-C stretching, C-H bending, and νs(PO_2−_) [65]. The second derivative spectra of these absorbance curves revealed that the absorbance at the minima positions at wavenumbers 1629 and 1652 cm^−1^ differ between healthy individuals and BC patients (Figure 2C). The elevation of absorbance values at the energy band 1018–1076 cm^−1^ (Figure 2D) suggests differences in the glycomic profiling [66] and circulating DNA [67] in the blood components. Circulating DNA and glycomic profiling have proven to be critical molecular markers [66,67] in several tumor entities.

### 2.3. Discrimination of Protein Secondary Structures

**Figure 3:**
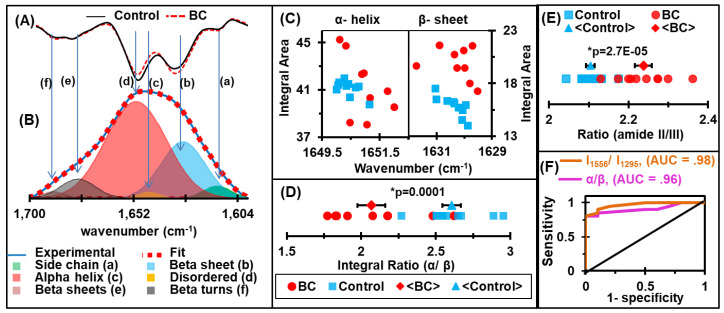
Protein secondary structure analysis. (**A**) Representative second derivatives of absorbance spectra at the amide-I absorbance region. (**B**) Deconvolution of the amide-I region: the baseline-corrected spectra fitted with 6 GFEB by approximating the number and position of the minima of second derivatives, which simulated fits (▪▪▪) to the experimental curve (—). (**C**) Integral area of GBEF representing α helix and β sheet. (**D**) The ratio of α helix and β sheet energy bands, which proves an elevation of β sheet and drop off α helix structures due to malignancies. (**E**) The ratio of IR absorbance at amide II (I_1556_) to its value at amide III (I_1295_). (**F**) Receiver Operating Characteristic (ROC) curves for the ratio of the integral area of the energy bands representing α-helix and β-sheet protein secondary structures and the respective absorbance at amide II and amide III. The maximum values of sensitivity and specificity are 90% and 90% for signature α/β, while these values are 100% and 80% for signature I_1556_/I_1295_, respectively.

In Figure 3A, the average of the second derivative spectra at the amide I absorbance region is shown. The minima of the second derivatives of the spectra allow us to approximate the positions and numbers of the Gaussian function energy profiles required to fit the experimental curve. The amide I band of each spectrum was deconvoluated so that the baseline-corrected spectra were fitted with six GFEB profiles by estimating the number and position of the minima of the second derivatives, which was simulated (**▪▪▪**) to fit the experimental curve (**—**), as shown in Figure 3B. Six Gaussian band profiles are assigned as (a) side chain (~1610 cm^−1^), (b) β sheet (~1630 cm^−1^), (c) random coil (~1645 cm^−1^), (d) α helix (~1652 cm^−1^), (e) β turn (~1682 cm^−1^), and (f) β anti-parallel sheet (~1690 cm^−1^) structures [75].

In order to assess any alterations in structural components associated with malignancy, the integral values of the α–helix and β–sheet structures and their ratios were analyzed. Due to the fact that the intensity of the GFEB has a linear relationship with the concentration according to the Beer–Lambert law [76], the width of GFEB and full width half maximum (FWHM) is inversely related to the vibrational mode lifetime, which is a function of the “rigidity” of the vibrating bond [35]. The interaction of the molecule with its immediate environment also affects the width of the GFEB [77]. If a molecule transfers energy to its surroundings, the spectral peak has a broader line width and reduced intensity, even though the concentration of the molecule remains unchanged. In such cases, the integral area under the curve is a better indicator of the concentration than the intensity alone. Interestingly, we found that even though the levels of most structures did not differ between the samples from the breast cancer patients and healthy individuals, the breast cancer samples had an increase in β–sheet structures, while the levels of the α-helix structures were decreased (Figure 3C,D). Furthermore, the amide II region is used to report on protein unfolding based on the extent of hydrogen exchanged. Because of the lack of water interference, the amide III region is also considered as a promising region to analyze protein structures. Herein, we have also used a ratio of IR absorbance at the amide II (I_1556_) to its value at the amide III (I_1295_) for the analysis of BC-associated protein alteration. The dot plots of these amides ratios are shown in Figure 3E.

### 2.4. Receiver Operating Characteristic (ROC) Curves and Area Under the Curve (AUC) Values

The sensitivity and specificity of a diagnostic test are often used to describe the diagnostic accuracy/performance of the analysis in biomedical research. The discriminating potential of a diagnostic regimen can be quantified by the AUC values of ROC curves [78]. The ROC curve is plotted to find the AUC, as in Figure 3F. The optimal cutoff value calculated for each spectral signature is used to select the positivity/negativity of the disease and to estimate the sensitivity and specificity. Strong discrimination between diseased and control serum can be seen with a 90% sensitivity and 90% specificity for signature α/β, and these values are 100% and 80% for I_1556_/ I_1295,_ respectively. The results indicated that the spectral signatures in the specified bands have a high diagnostic accuracy.

**Figure 4:**
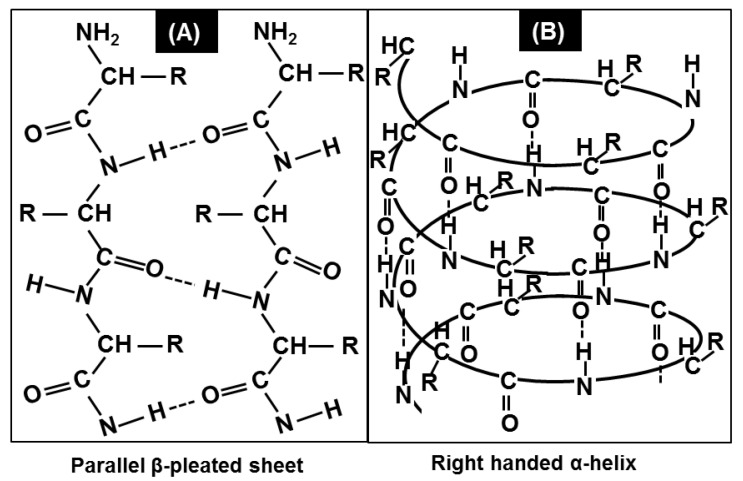
Understanding the protein secondary structures and physics of IR interaction. (**A**) Parallel β-pleated sheet structure of proteins. N-H groups in the backbone of one strand form hydrogen bonds with the C=O groups in the backbone of the adjacent strand to form a β-sheet. (**B**) Right-handed α-helix structures of proteins. The backbone N-H group donates a hydrogen bond to the backbone C=O group, contributing to the helical structure of the α-helix.

As shown in Figure 4, the backbone N-H group donates a hydrogen bond to the backbone C=O group to form the helical structure of the α-helix (Figure 4B). In contrast, the backbone N-H groups of one strand can form hydrogen bonds with the backbone C=O groups of the adjacent strands, resulting in β-sheet structures (Figure 4A). Therefore, the cancer-associated alterations in the integral ratio of α-helix and β-sheet protein secondary structures suggest that protein conformational alterations accompanying changes in their biological function might be a key event during the development of cancer. Several studies have shown that the proteins in serum change during breast cancer [21,22,23,79]. The alterations in the conformational compositions are presumably due to alteration in the concentration of cancer embryonic antigen (CEA) proteins [7].

## 3. Discussion

Protein analysis is considered as a promising technique for understanding the progression of cancers. Similarly, FTIR spectral analysis is one of the accepted paradigms for the holistic evaluation of protein structural content at the molecular level in biological samples. Several studies have introduced the applicability of FTIR spectroscopy in serum samples accompanied by spectral analysis techniques for BC discrimination [22,23,29,80,81]. Reports [22,29] show the potential application of FTIR spectroscopy for protein analysis in the serum samples from BC patients. However, cancer initiation, progression, and response to therapy depend on an array of complex interactions between constituent biomolecules (proteins, lipids, nucleic acids, and carbohydrates) and not only at the level of the single (biomarker or target) molecule. Therefore, the feasibility of FTIR spectroscopy to extract a snapshot of cumulative molecular interactions within serum samples warrants a thorough investigation, as enabled by interdisciplinary collaboration between spectroscopists, biologists, and clinicians. It is noted that the evaluation of serological biomarkers (CA15-3, HSP90A, and PAI-1) do not show consistent differences between BC cases and controls that can lead to diagnosis [7]. Our data show alterations in the biochemical, and structural, information of the constituent components of the sample medium. As such, the holistic evaluation of biochemical details with the use of infrared spectroscopy can thus have an immense potential for BC discrimination analysis in the clinical domain.

### 3.1. Deconvolution of Spectral Range 1140–1000 cm^−1^

**Figure 5:**
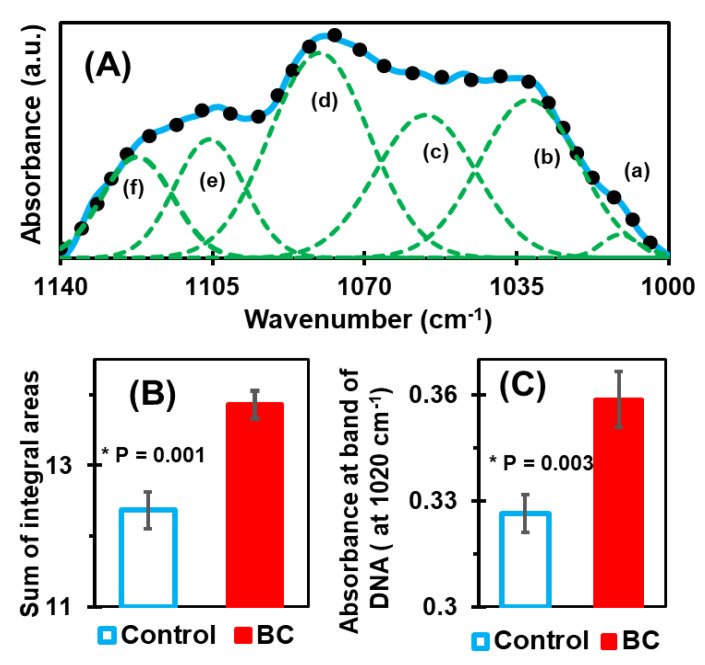
(**A**) Deconvolution of the complex band of carbohydrates and nucleic acids at 1000–1140 cm^−1^. The number and position of the six bands used to fit the experimental curve were determined by using the minima of secondary curves, as in the amide I case. (**B**) Bar graph representation of the average value of the integral sum, which shows a significant difference between the control and BC case. (**C**) Bar graph of the average absorbances at wavenumber position 1020 cm^−1^, which is mainly due to the presence of DNA. It also shows a significant difference between the control and BC cases.

To analyze the snapshot of alterations reflected in our FTIR spectral data, the complex region [62] of carbohydrates and nucleic acids, 1140–1000 cm^−1^, was deconvoluted. The BC-associated alterations in the DNA and RNA are reflected in this region. Circulating DNA and protein markers are generally evaluated to track the biomolecular events of cancerous patients [82]. Herein, this spectral range is deconvoluted with six GFEB (Figure 5A) by approximating the numbers and positions using the minima of second derivatives. The sum of integral areas covered by six bands (integral values) of control, samples range from 11.4 to 13.2, while these values in BC samples are from 13 to 14.8. This quantified information was further statistically analyzed (Figure 5B), and the result shows a clear separation between control and BC. Similarly, Figure 5C shows the histogram of the average values of absorbance at wavenumber 1020 cm^−1^. The absorbance at this energy band is found to be due to the presence of circulating DNA [61,62].

### 3.2. Potential Prototype for Clinical Application

**Figure 6:**
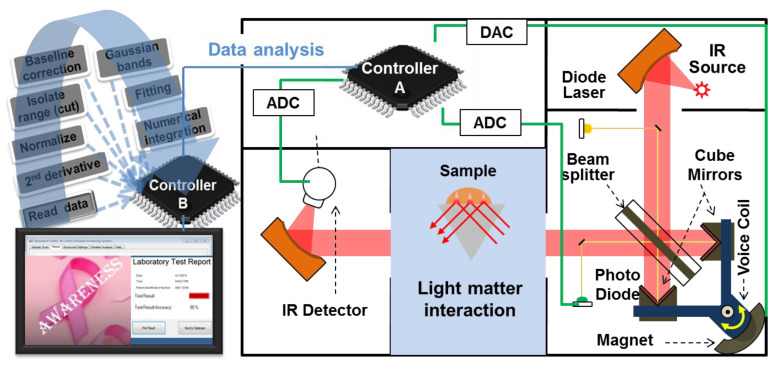
Schematic of attenuated total reflection Fourier transforms infrared (ATR-FTIR) spectrometer integrated with two micro-controllers (micro-processors) A and B. Controller A extracts the information about the signal–sample interaction, while controller B stores the spectral analyzing software application in the clinical domain.

Moreover, a prototype for our presented diagnostic regimen for clinical use can be developed. Spectral measurements and data analysis procedures will be automated into a single step so that a technician can deposit the sample on to the sample holder and start to measure with a simple click to get the result and, if needed, the biochemical information easily as shown in Figure 6. Here, attenuated total reflection Fourier transforms infrared (ATR-FTIR) spectroscopy (that is reliable for body-fluids analysis) integrated with two micro-controllers, where micro-controller A controls all the functions in the FTIR and extracts information about signal sample interaction, while the controller B controls software for data analysis. The software program will include several subroutines as reading spectral data from the FTIR; extract data for suitable spectral signatures in the measured range; normalizing and baseline correction of spectral data subroutines will have simple loops, condition checks, and basics mathematical calculations. The second derivative will be calculated by using divided difference formulas for discrete data. After finding various minimums and their positions, the program will assign parameters for Gaussian energy bands and select settings for bands to minimize RMS error (Levenberg Marquardt algorithm) between the experimental data and fitted curves. The standard numerical integration technique will be used to find the area under Gaussian bands and the ratio. Additionally, combining all the identified multiple spectral signatures into a single diagnostic index using them as the discriminating signature marker, a portable device integrated with the user-friendly desktop unit (can automatically perform the full data analysis and will display laboratory test report) can be prepared.

All in all, the FTIR spectroscopy of serum samples could be a promising technique for an Affordable, Sensitive, Specific, User-friendly, Robust and rapid, Equipment-free, and Deliverable (ASSURED) regimen for the evaluation of BC-associated molecular level of alteration in constituent protein structures. Our study holds value, as available techniques such as mammograms, MRI, and ultrasonography have their limits and may not be 100% accurate [4,83,84,85]. Among them, MRI achieves a high sensitivity of 70–100% in the initial screening (prevalence), compared at 40% or less for mammography in patients with high risk to develop BC [84,85], but the specificity of MRI is hampered by its difficulty while distinguishing the overlapping features of benign and malignant lesions, leading to higher false-positive rates [83]. Ultrasonography also fails to detect micro-calcifications and has a poor specificity. Therefore, the present diagnostic regimen of BC having the potential to promote timely onward referral of patients for further testing and detection of recurrent disease, “enabling serial sample and testing with less cost, resource and radiation exposure” could be beneficial for several patients.

## 4. Materials and Methods

### 4.1. Human Sera

Human sera from breast cancer patients were obtained from the Breast Satellite Tissue Bank, Winship Cancer Institute, Emory University, Atlanta GA, USA. The Helsinki Declaration guidelines were followed for sample collection, and informed consent was obtained from all the patients (females, age 30–65 years, see Table S1 for details). Blood was collected without additives from patients after informed consent. The blood was then centrifuged at ~3200g for 10 min, and the serum was pipetted and stored at -80^o^C until analysis. The control healthy individual sera were from the baseline collection of healthy women (age 41–58 years) participating in an independent intervention study under approval number 13317, Edith Cowan University, Perth, Australia. All the participants provided informed consent. The sera were thawed, aliquoted in small volumes, and stored at −80 ^o^C until analysis.

### 4.2. FTIR Spectrometer

Spectral data were obtained using a Bruker Vertex-70 FTIR spectrometer fitted with a potassium bromide (KBr) beam splitter and Deuterated Tri-Glycine Sulfate (DTGS) detector. Furthermore, this study utilized an MVP-Pro ATR accessory fitted with a diamond crystal, which was configured to have a single reflection. To achieve the best resolution available, a Medium Blackman–Harris apodization function was used with a resolution of 4 cm^−1^ and a zero-filling factor of four. This choice was opted for because a weak apodization leads to a higher resolution, but at the cost of increasing noise. Typically, a medium apodization is recommended [86] for liquids, gels, and semi-solids, such as the Blackman–Harris three-term used in this study. The aperture size was set to 2.5 mm for the optimization of the detector response without saturation.

### 4.3. ATR-FTIR Spectral Measurements

To get rid of excess staining substances, the ATR crystal surface (in the FTIR light path) was thoroughly cleaned with sterile phosphate-buffered saline and ethanol before use. Sufficient cleanliness was confirmed by ensuring that the absorbance spectrum obtained without a sample contained no peaks higher than the noise level. Prior to each spectral scanning, a background measurement was performed by collecting data from the cleaned crystal surface and subtracting it from the sample signal spectrum. One microliter of each sample was deposited, allowed to settle to room temperature (~4 min), then scanned multiple times to yield high-quality, reproducible spectra. Variations due to moisture were avoided by drying the serum samples, as described previously [46].

### 4.4. Spectral Analysis

Each sample was scanned within the range of 400 to 4000 cm^−1^ until at least eight high-quality spectral curves were obtained. Further statistical analysis was carried out on an average of 100 co-added scans for each sample. A total of 20 spectra of serums (representing 20 individuals; control 10 and BC 10) were obtained. However, to perform a multivariate analysis a repeated measurement was performed on each sample (to get 20 spectra of control and 20 spectra of BC). For the multivariate analysis, the second derivatives curves of the absorbance spectra were vector-normalized, while throughout other studies the spectral data (absorption spectra) were min-max normalized [13] using the OPUS 6.5 software within the fingerprint region of 1800 to 900 cm^−1^. The absorbance value of the amide I band position (~1642 cm^−1^) was 2 AU, corresponding to ~99% absorption according to the Beer–Lambert law [76]. Herein, we have selected the spectral region of 1800 to 900 cm^−1^ by avoiding the strong moisture absorption region as suggested. We have then studied the settling and uniformity while drying the serum samples in the crystal surface to determine the optimum settling time considering the reproducibility of curves. The spectral region of 1800 to 900 cm^−1^ shows excellent reproducibility, so the proteomic signatures reflected in this range are only analyzed in this study.

The complex IR spectral bands of amides I band were deconvoluted into six GFEBs, which involved the normalization of spectra, the sectioning of the necessary bands, and baseline rubber-band correction so that the absorbance at the endpoints becomes zero. The complex region of carbohydrates and nucleic acids, 1140–1000 cm^−1^, was further deconvoluted into six GFEBs. The position and number of GFEB used to fit the experimental curves were determined using the minima of the original spectra’s second derivatives. By utilizing the Levenberg–Marquardt algorithm, the experimental curves were fitted, minimizing the RMS error, which is indicative of successful [56] GFEB fitting.

## 5. Conclusions

The ratio of the integral areas of GFEB representing α-helix and β-sheet protein secondary structures (α/β) and absorbance values (I_1556_/I_1295_) are identified as unique proteomics spectral signatures for BC discrimination. The discriminating potential of the technique, as well as its sensitivity and specificity, were further assessed using the AUC values of ROC curves (Table 2). The maximum values of sensitivity and specificity for each feature describe the difference between the BC and control sera. The AUC, sensitivity, and specificity values for the α-helix/β-sheet ratio were 0.955, 80%, and 100%, respectively, with a *p*-value of 1.4E-04, while for the I_1556_/ I_1295_ ratio these values were 0.98, 100%, and 80%, respectively, with a *p*-value of 2.7 × 10^−5^.

In conclusion, this study provides evidence that the BC-associated proteomics conformational changes in the serum samples of BC patients can be analyzed by using a curve-fitting technique of infrared spectral data. The study also provides a detailed insight into the protein structure changes that occur in BC patients, paving the way for further large-scale studies. The detailed study presented a new regimen of BC discrimination which would allow the assessment of disease status and therapeutic efficacy. In this study, the possible discrimination of nucleic acids and carbohydrate regions by using curve fitting is also presented. The simultaneous fitting of these absorption bands provides a more robust base for the structural studies of proteins and complex band contours. In addition, future research directions are also presented. The potential use of the spectrometric assessment of serum protein conformation in breast cancer diagnosis and monitoring, as well as the relevance of serum protein conformational changes in cancer development, merit further investigation towards establishing a successful clinical technique in the future.

## Figures and Tables

**Table 1:** Discriminatory IR bands for BC serum samples from controls, and primary biomolecular assignments giving rise to the main contributions for the absorbance (taken from [68,69,70,71,72,73,74]). Amide regions and the complex region of carbohydrates and nucleic acids show the discriminating potential.

Wavenumber (cm^−1^)	Biomolecular Assignments
1700–1600	Amide I: sensitive to protein secondary structures of proteins, which arises mainly due to C=O stretching vibrations and the C-N groups.
1580–1480	Amide II: sensitive for protein conformation, originates mainly from the in-plane N-H bending mode along with C-N and C-C stretching vibrations.
1140–1000	Carbohydrates: sensitive to C-O, C-C stretching, C-H bending, and nucleic acids: sensitive to deoxyribose/ribose DNA, RNA, ν_s_(PO_2_^−^).

**Table 2:** Identifying BC-associated discriminatory protein bands in serum samples. These include the integral ratio of Gaussian Function Energy Bands (GFEB), representing α-helix and β-sheet protein secondary structures, as well as the absorbance ratio of amide II (1556 cm^−1^) to amide III (1295 cm^−1^). Quantified values (in A.U.) of the average and range of spectral signatures taken from the control and BC samples. The optimal cutoff and the corresponding sensitivity, specificity, and *p*-values are also shown.

Signatures	Average ± st. Error		Range of Values		Cutoff Value	AUC	Sensitivity %	Specificity %	*p*-value
	Control	BC	Control	BC					
α/β	2.61 ± 0.06	2.07 ± 0.09	2.27–2.95	1.77–2.62	2.25	0.96	90	90	1.4 × 10^−4^
I_1556_/ I_1295_	2.10 ± 0.01	2.24 ± 0.02	2.04–2.17	2.13–2.36	2.12	0.98	100	80	2.7× 10^−5^

## Supplementary Materials

The following are available online at https://www.mdpi.com/2072-6694/12/7/1708/s1: Table S1: Breast cancer (BC) patients’ information.

## References

[1] Stewart B., Wild C.P. (2019). World cancer report 2014. Public Health 2019.

[2] McGuire S. (2016). World Cancer Report 2014. Geneva, Switzerland: World Health Organization, International Agency for Research on Cancer, WHO Press, 2015.

[3] Society A.C. (2017). Breast Cancer Facts & Figures 2017–2018.

[4] Yankaskas B.C., Haneuse S., Kapp J.M., Kerlikowske K., Geller B., Buist D.S. (2010). Performance of first mammography examination in women younger than 40 years. J. Natl. Cancer Inst..

[5] Miller A.B., Wall C., Baines C.J., Sun P., To T., Narod S.A.J.B. (2014). Twenty five year follow-up for breast cancer incidence and mortality of the Canadian National Breast Screening Study: Randomised screening trial. BMJ.

[6] Misek D.E., Kim E.H. (2011). Protein biomarkers for the early detection of breast cancer. Int. J. Proteom..

[7] Kazarian A., Blyuss O., Metodieva G., Gentry-Maharaj A., Ryan A., Kiseleva E.M., Prytomanova O.M., Jacobs I.J., Widschwendter M., Menon U. (2017). Testing breast cancer serum biomarkers for early detection and prognosis in pre-diagnosis samples. Br. J. Cancer.

[8] Blanco M., Villarroya I. (2002). NIR spectroscopy: A rapid-response analytical tool. TrAC Trends Anal. Chem..

[9] Kendall C., Isabelle M., Bazant-Hegemark F., Hutchings J., Orr L., Babrah J., Baker R., Stone N. (2009). Vibrational spectroscopy: A clinical tool for cancer diagnostics. Analyst.

[10] Dubois J., Shaw R.A. (2004). Peer Reviewed: IR Spectroscopy in Clinical and Diagnostic Applications.

[11] Parker F. (2012). Applications of Infrared Spectroscopy in Biochemistry, Biology, and Medicine.

[12] Movasaghi Z., Rehman S., ur Rehman D.I. (2008). Fourier transform infrared (FTIR) spectroscopy of biological tissues. Appl. Spectrosc. Rev..

[13] Baker M.J., Trevisan J., Bassan P., Bhargava R., Butler H.J., Dorling K.M., Fielden P.R., Fogarty S.W., Fullwood N.J., Heys K.A. (2014). Using Fourier transform IR spectroscopy to analyze biological materials. Nat. Protoc..

[14] Yang H., Yang S., Kong J., Dong A., Yu S. (2015). Obtaining information about protein secondary structures in aqueous solution using Fourier transform IR spectroscopy. Nat. Protoc..

[15] Trevisan J., Angelov P.P., Carmichael P.L., Scott A.D., Martin F.L. (2012). Extracting biological information with computational analysis of Fourier-transform infrared (FTIR) biospectroscopy datasets: Current practices to future perspectives. Analyst.

[16] Kelly J.G., Angelov P.P., Trevisan J., Vlachopoulou A., Paraskevaidis E., Martin-Hirsch P.L., Martin F.L. (2010). Robust classification of low-grade cervical cytology following analysis with ATR-FTIR spectroscopy and subsequent application of self-learning classifier eClass. Anal. Bioanal. Chem..

[17] Eckel R., Huo H., Guan H.W., Hu X., Che X., Huang W. (2001). Characteristic infrared spectroscopic patterns in the protein bands of human breast cancer tissue. Vib. Spectrosc..

[18] Blobe G.C., Obeid L.M., Hannun Y.A. (1994). Regulation of protein kinase C and role in cancer biology. Cancer Metastasis Rev..

[19] Bartkova J., Lukas J., Müller H., Lützhøt D., Strauss M., Bartek J. (1994). Cyclin D1 protein expression and function in human breast cancer. Int. J. Cancer.

[20] Jacquemier J., Ginestier C., Rougemont J., Bardou V.J., Charafe-Jauffret E., Geneix J., Adélaïde J., Koki A., Houvenaeghel G., Hassoun J. (2005). Protein expression profiling identifies subclasses of breast cancer and predicts prognosis. Cancer Res..

[21] Li J., Zhang Z., Rosenzweig J., Wang Y.Y., Chan D.W. (2002). Proteomics and bioinformatics approaches for identification of serum biomarkers to detect breast cancer. Clin. Chem..

[22] Elmi F., Movaghar A.F., Elmi M.M., Alinezhad H., Nikbakhsh N. (2017). Application of FT-IR spectroscopy on breast cancer serum analysis. Spectrochim. Acta Part A Mol. Biomol. Spectrosc..

[23] Zelig U., Barlev E., Bar O., Gross I., Flomen F., Mordechai S., Kapelushnik J., Nathan I., Kashtan H., Wasserberg N. (2015). Early detection of breast cancer using total biochemical analysis of peripheral blood components: A preliminary study. BMC Cancer.

[24] Ostrovsky E., Zelig U., Gusakova I., Ariad S., Mordechai S., Nisky I., Kapilushnik J. (2012). Detection of cancer using advanced computerized analysis of infrared spectra of peripheral blood. IEEE Trans. Biomed. Eng..

[25] Gao T., Feng J., Ci Y. (1999). Human breast carcinomal tissues display distinctive FTIR spectra: Implication for the histological characterization of carcinomas. Anal. Cell. Pathol..

[26] Lyman D.J., Murray-Wijelath J. (2005). Fourier transform infrared attenuated total reflection analysis of human hair: Comparison of hair from breast cancer patients with hair from healthy subjects. Appl. Spectrosc..

[27] Han S.M., Chikawa J.I., Jeon J.K., Hwang M.Y., Lim J., Jeong Y.J., Park S.H., Kim H.T., Jheon S., Kim J.K. (2016). Synchrotron nanoscopy imaging study of scalp hair in breast cancer patients and healthy individuals: Difference in medulla loss and cortical membrane enhancements. Microsc. Res. Tech..

[28] Malins D.C., Polissar N.L., Schaefer S., Su Y., Vinson M. (1998). A unified theory of carcinogenesis based on order–disorder transitions in DNA structure as studied in the human ovary and breast. Proc. Natl. Acad. Sci. USA.

[29] Kotkova M., Sitnikova V., Nosenko T., Kotkova T., Uspenskaya M., Olekhnovich R. Spectroscopic Study of Blood Serum of Patients with Breast Cancer. Proceedings of the 2018 IEEE-EMBS Conference on Biomedical Engineering and Sciences (IECBES).

[30] Mitchell A.L., Gajjar K.B., Theophilou G., Martin F.L., Martin-Hirsch P.L. (2014). Vibrational spectroscopy of biofluids for disease screening or diagnosis: Translation from the laboratory to a clinical setting. J. Biophotonics.

[31] Aaboe M., Offersen B.V., Christensen A., Andreasen P.A. (2003). Vitronectin in human breast carcinomas. Biochim. Biophys. Acta Mol. Basis Dis..

[32] Karplus M., McCammon J.A. (2002). Molecular dynamics simulations of biomolecules. Nat. Struct. Mol. Biol..

[33] Drenth J. (2007). Principles of Protein X-Ray Crystallography.

[34] Wüthrich K. (1986). NMR with proteins and nucleic acids. Europhys. News.

[35] Barth A. (2007). Infrared spectroscopy of proteins. Biochim. Biophys. Acta.

[36] Jabs A.J. (2005). Determination of Secondary Structure in Proteins by Fourier Transform Infrared Spectroscopy (FTIR).

[37] Titus J., Ghimire H., Viennois E., Merlin D., Perera A.G. (2018). Protein secondary structure analysis of dried blood serum using infrared spectroscopy to identify markers for colitis screening. J. Biophotonics.

[38] Hering J.A., Haris P.I. (2009). FTIR spectroscopy for analysis of protein secondary structure. Biol. Biomed. Infrared Spectrosc..

[39] Surewicz W.K., Mantsch H.H., Chapman D. (1993). Determination of protein secondary structure by Fourier transform infrared spectroscopy: A critical assessment. Biochemistry.

[40] Lee D.C., Haris P.I., Chapman D., Mitchell R.C. (1990). Determination of protein secondary structure using factor analysis of infrared spectra. Biochemistry.

[41] Pribic R., Vanstokkum I., Chapman D., Haris P.I., Bloemendal M. (1993). Protein secondary structure from Fourier transform infrared and/or circular dichroism spectra. Anal. Biochem..

[42] Miller L.M., Bourassa M.W., Smith R.J. (2013). FTIR spectroscopic imaging of protein aggregation in living cells. Biochim. Biophys. Acta Biomembr..

[43] Haris P.I., Severcan F. (1999). FTIR spectroscopic characterization of protein structure in aqueous and non-aqueous media. J. Mol. Catal. B Enzym..

[44] Garczarek F., Gerwert K. (2006). Functional waters in intraprotein proton transfer monitored by FTIR difference spectroscopy. Nature.

[45] Grdadolnik J., Maréchal Y. (2005). Hydrogen–Deuterium Exchange in Bovine Serum Albumin Protein Monitored by Fourier Transform Infrared Spectroscopy, Part I: Structural Studies. Appl. Spectrosc..

[46] Prestrelski S.J., Tedeschi N., Arakawa T., Carpenter J.F. (1993). Dehydration-induced conformational transitions in proteins and their inhibition by stabilizers. Biophys. J..

[47] Lu R., Li W.W., Katzir A., Raichlin Y., Yu H.Q., Mizaikoff B. (2015). Probing the secondary structure of bovine serum albumin during heat-induced denaturation using mid-infrared fiberoptic sensors. Analyst.

[48] Fabian H., Schultz C., Naumann D., Landt O., Hahn U., Saenger W. (1993). Secondary structure and temperature-induced unfolding and refolding of ribonuclease T1 in aqueous solution: A Fourier transform infrared spectroscopic study. J. Mol. Biol..

[49] Baker M.J., Gazi E., Brown M.D., Shanks J.H., Gardner P., Clarke N.W. (2008). FTIR-based spectroscopic analysis in the identification of clinically aggressive prostate cancer. Br. J. Cancer.

[50] Ghimire H., Venkataramani M., Bian Z., Liu Y., Perera A.U. (2017). ATR-FTIR spectral discrimination between normal and tumorous mouse models of lymphoma and melanoma from serum samples. Sci. Rep..

[51] Choo L.P.I., Wetzel D.L., Halliday W.C., Jackson M., LeVine S.M., Mantsch H.H. (1996). In situ characterization of beta-amyloid in Alzheimer’s diseased tissue by synchrotron Fourier transform infrared microspectroscopy. Biophys. J..

[52] Szczerbowska-Boruchowska M., Dumas P., Kastyak M.Z., Chwiej J., Lankosz M., Adamek D., Krygowska-Wajs A. (2007). Biomolecular investigation of human substantia nigra in Parkinson’s disease by synchrotron radiation Fourier transform infrared microspectroscopy. Arch. Biochem. Biophys..

[53] Kretlow A., Wang Q., Beekes M., Naumann D., Miller L.M. (2008). Changes in protein structure and distribution observed at pre-clinical stages of scrapie pathogenesis. Biochim. Biophys. Acta Mol. Basis Dis..

[54] Haris P.I. (2013). Probing protein–protein interaction in biomembranes using Fourier transform infrared spectroscopy. Biochim. Biophys. Acta Biomembr..

[55] Jackson M., Haris P.I., Chapman D. (1991). Fourier transform infrared spectroscopic studies of calcium-binding proteins. Biochemistry.

[56] Jackson M., Mantsch H.H. (1995). The use and misuse of FTIR spectroscopy in the determination of protein structure. Crit. Rev. Biochem. Mol. Biol..

[57] Surewicz W.K., Mantsch H.H. (1988). New insight into protein secondary structure from resolution-enhanced infrared spectra. Biochim. Biophys. Acta Protein Struct. Mol. Enzymol..

[58] Fabian H., Naumann D. (2004). Methods to study protein folding by stopped-flow FT-IR. Methods.

[59] Zhang S., Rich A. (1997). Direct conversion of an oligopeptide from a β-sheet to an α-helix: A model for amyloid formation. Proc. Natl. Acad. Sci. USA.

[60] Kong J., Yu S. (2007). Fourier transform infrared spectroscopic analysis of protein secondary structures. Acta Biochim. Biophys. Sin..

[61] Dovbeshko G.I., Gridina N.Y., Kruglova E.B., Pashchuk O.P. (2000). FTIR spectroscopy studies of nucleic acid damage. Talanta.

[62] Sahu R., Argov S., Salman A., Huleihel M., Grossman N., Hammody Z., Kapelushnik J., Mordechai S. (2004). Characteristic absorbance of nucleic acids in the Mid-IR region as possible common biomarkers for diagnosis of malignancy. Technol. Cancer Res. Treat..

[63] Wold S., Esbensen K., Geladi P. (1987). Principal component analysis. Chemom. Intell. Lab. Syst..

[64] Ollesch J., Drees S.L., Heise H.M., Behrens T., Brüning T., Gerwert K. (2013). FTIR spectroscopy of biofluids revisited: An automated approach to spectral biomarker identification. Analyst.

[65] Lovergne L., Clemens G., Untereiner V., Lukaszweski R.A., Sockalingum G.D., Baker M.J. (2015). Investigating optimum sample preparation for infrared spectroscopic serum diagnostics. Anal. Methods.

[66] Ghazarian H., Idoni B., Oppenheimer S.B. (2011). A glycobiology review: Carbohydrates, lectins and implications in cancer therapeutics. Acta Histochem..

[67] Heitzer E., Ulz P., Geigl J.B. (2015). Circulating tumor DNA as a liquid biopsy for cancer. Clin. Chem..

[68] Meurens M., Wallon J., Tong J., Noel H., Haot J. (1996). Breast cancer detection by Fourier transform infrared spectrometry. Vib. Spectrosc..

[69] Baker M.J., Hussain S.R., Lovergne L., Untereiner V., Hughes C., Lukaszewski R.A., Thiéfin G., Sockalingum G.D. (2016). Developing and understanding biofluid vibrational spectroscopy: A critical review. Chem. Soc. Rev..

[70] Gazi E., Baker M., Dwyer J., Lockyer N.P., Gardner P., Shanks J.H., Reeve R.S., Hart C.A., Clarke N.W., Brown M.D. (2006). A correlation of FTIR spectra derived from prostate cancer biopsies with Gleason grade and tumour stage. Eur. Urol..

[71] Gajjar K., Heppenstall L.D., Pang W., Ashton K.M., Trevisan J., Patel I.I., Llabjani V., Stringfellow H.F., Martin-Hirsch P.L., Dawson T. (2013). Diagnostic segregation of human brain tumours using Fourier-transform infrared and/or Raman spectroscopy coupled with discriminant analysis. Anal. Methods.

[72] Hands J.R., Clemens G., Stables R., Ashton K., Brodbelt A., Davis C., Dawson T.P., Jenkinson M.D., Lea R.W., Walker C. (2016). Brain tumour differentiation: Rapid stratified serum diagnostics via attenuated total reflection Fourier-transform infrared spectroscopy. J. Neuro-Oncol..

[73] Hands J.R., Dorling K.M., Abel P., Ashton K.M., Brodbelt A., Davis C., Dawson T., Jenkinson M.D., Lea R.W., Walker C. (2014). Attenuated total reflection Fourier transform infrared (ATR-FTIR) spectral discrimination of brain tumour severity from serum samples. J. Biophotonics.

[74] Krimm S., Bandekar J. (1986). Vibrational spectroscopy and conformation of peptides, polypeptides, and proteins. Advances in Protein Chemistry.

[75] Goormaghtigh E., Ruysschaert J.M., Raussens V. (2006). Evaluation of the information content in infrared spectra for protein secondary structure determination. Biophys. J..

[76] Swinehart D.J. (1962). The beer-lambert law. J. Chem. Educ..

[77] Schweizer K.S., Chandler D. (1982). Vibrational dephasing and frequency shifts of polyatomic molecules in solution. J. Chem. Phys..

[78] Šimundić A.M. (2009). Measures of diagnostic accuracy: Basic definitions. Ejifcc.

[79] Gast M.C.W., Van Gils C.H., Wessels L.F., Harris N., Bonfrer J.M., Rutgers E.J., Schellens J.H., Beijnen J.H. (2009). Serum protein profiling for diagnosis of breast cancer using SELDI-TOF MS. Oncol. Rep..

[80] Backhaus J., Mueller R., Formanski N., Szlama N., Meerpohl H.G., Eidt M., Bugert P. (2010). Diagnosis of breast cancer with infrared spectroscopy from serum samples. Vib. Spectrosc..

[81] Andrei A.B., Fleschin Ş., Aboul-Enein H.Y. (2015). Cancer diagnosis by FT-IR Spectrophotometry. Rev. Roum. Chim..

[82] Schwarzenbach H., Pantel K. (2015). Circulating DNA as biomarker in breast cancer. Breast Cancer Res..

[83] Kriege M., Brekelmans C.T., Obdeijn I.M., Boetes C., Zonderland H.M., Muller S.H., Kok T., Manoliu R.A., Besnard A.P.E., Tilanus-Linthorst M.M. (2006). Factors affecting sensitivity and specificity of screening mammography and MRI in women with an inherited risk for breast cancer. Breast Cancer Res. Treat..

[84] Kriege M., Brekelmans C.T.M., Boetes C., Besnard P.E., Zonderland H.M., Obdeijn I.M., Manoliu R.A., Kok T., Peterse H., Tilanuslinthorst M.M.A. (2004). Efficacy of MRI and mammography for breast-cancer screening in women with a familial or genetic predisposition. N. Engl. J. Med..

[85] Leach M.O., Boggis C., Dixon A.K., Easton D.F., Eeles R.A., Evans D.G.R., Gilbert F.F., Griebsch I., Hoff R., Kessar P. (2005). Screening with magnetic resonance imaging and mammography of a UK population at high familial risk of breast cancer: A prospective multicentre cohort study (MARIBS). Lancet.

[86] Naylor D.A., Tahic M.K. (2007). Apodizing functions for Fourier transform spectroscopy. J. Opt. Soc. Am. A.

