# Supplementary material for: Protein Conformational Changes in Breast Cancer Sera Using Infrared Spectroscopic Analysis
Source: Cancers (Basel). 2020 Jun 27;12(7):1708. doi: 10.3390/cancers12071708 (PMC7407230; doi:10.3390/cancers12071708)
Supplement: Supplementary file 1 [file cancers-12-01708-s001.pdf]

cancers

Protein Conformational Changes in Breast Cancer Sera Using Infrared Spectroscopic Analysis

Hemendra Ghimire, Chakravarthy Garlapati, Emiel A. M. Janssen, Uma Krishnamurti, Gengsheng Qin, Ritu Aneja, and A. G. Unil Perera

Supporting Information

Breast cancer (BC) patients' information

Table S1. Information about the patients: Biopsy results such as Invasive lobular carcinoma (ILC), Invasive ductal carcinoma (IDC), Ductal Carcinoma In-Situ (DCIS) confirmed the BC diagnoses. The tumor grading assessment was performed based on the national cancer institute (NCI) guidelines. Grade-2 denotes the intermediate grade or moderately differentiated tumor where as grade-3 denotes a high grade or poorly differentiated tumor.

ID	Age years	BMI	Menstrual Status	BC Discovery	History	Tumor Size	Laterality Biopsy	Biopsy Results
1	41.95	40.09	Pre menopausal	Screening	No	.9 x .9 x 1.1cm	Right	ILC
2	56.55	28.63	Post menopausal	Screening	Yes	1 x .8cm	Right	IDC
3	65.36	33.43	Post menopausal	Accidental	No	1.99x1.82 x1.14cm	Left	IDC
4	46.98	38.52	N/A	Accidental	No	5.3x3.9 cm	Right	IDC
5	50.39	54.8	N/A	Screening	No	2.5 cm	Right	IDC
6	37.86	N/A	Pre menopausal	Accidental	No	6.6 x 3.9 x 4.2 cm	Left	IDC
7	58.24	21.14	Unknown	Self-exam	Yes	5 cm	Left	IDC
8	30.97	33.59	Pre menopausal	Self-exam	No	2.4 x 2.1 cm	Right	IDC
9	65.97	25.96	Post menopausal	Self-exam	No	5.0 cm	Left	IDC
10	63.75	22.78	N/A	Pain	Yes	7.0 x 5.3 x 3.7 cm	Left	IDC

Type of Surgery	Lymph Nodes	Malignant Grade
Mastectomy	Yes	2
Mastectomy SLNB	Yes	3
Mastectomy	Yes	3
Partial Mastectomy (lumpectomy) SLNB	No	3
Mastectomy ALND	Yes	3
Mastectomy SLNB	No	3
Mastectomy SLNB	No	3
Partial Mastectomy (lumpectomy) SLNB	Yes	3
Partial Mastectomy (lumpectomy) SLNB	No	2
Mastectomy SLNB	No	3

Table S1. shows the breast cancer (BC) patients clinical information. The age of BC patients ranges from 30 years to 65 years and the age of control volunteers lies within this interval (41 to 58 years). The median age for BC patients and control volunteers remains similar; 53 years for BC and 52 years for controls. All BC patients were triple-negative (the hormone receptor subtype; estrange receptor (ER), progensterone receptor (PR) and human epidermal growth factor receptor-2 (HER2/neu) status was negative).
